# Congenital nystagmus in two infants born from mothers exposed to methadone during pregnancy

**DOI:** 10.1186/1824-7288-39-40

**Published:** 2013-07-03

**Authors:** Francesca Tinelli, Alessandra Gamucci, Roberta Battini, Giovanni Cioni

**Affiliations:** 1 Department of Developmental Neuroscience, IRCCS Fondazione Stella Maris, Via dei Giacinti 2, Calambrone, Pisa 56128, Italy; 2 Department of Clinical and Experimental Medicine, University of Pisa, Pisa, Italy

**Keywords:** Congenital nystagmus, Methadone, Pregnancy

## Abstract

**Background:**

Methadone is commonly prescribed as a substitute for illicit opioids. Use of methadone during pregnancy is associated with neonatal abstinence syndrome (NAS), reduced head circumference as well as a slight increase in neonatal mortality and morbidity. Less is known about the effects of methadone on the visual system.

**Cases:**

We report two Italian cases of nystagmus in infants born from mothers exposed to methadone during pregnancy. Ophthalmic or central disorders were excluded as a cause of nystagmus in both infants. The first case was followed at 3, 6 and 12 months while the second one was evaluated at 5 and 8 months. Both infants had normal neurological and cognitive development. Their first evaluation revealed different characteristics but both showed progressive improvement in ocular disorder, persistence of pendular horizontal nystagmus and nearly normal visual acuity.

**Conclusion:**

This report, the first description of Italian cases of nystagmus related to use of methadone during pregnancy, underlies the importance of a careful investigation of drug use in pregnancy in cases of unexplained congenital nystagmus.

## Background

Methadone is commonly prescribed as a substitute for illicit opioids. It is a synthetic opioid which selectively binds to the μ-opioid receptor, exerting a morphine-like effect [1]. Use of methadone during pregnancy is associated with reduced head circumference [2,3] as well as slight increases in neonatal mortality [4-6] and morbidity and often neonatal abstinence syndrome (NAS) [7-9]. Less is known about the effects of methadone on visual system. Only a few studies [10-14] have been published on this topic which evidenced reduced acuity, nystagmus, delayed visual maturation and eye movement disorders in children born from mothers exposed to methadone during pregnancy, even if fundus examination was normal. Recently Cornish et al. [15] published a study on the short and long term effects on the visual system of children exposed to maternal drug misuse *in utero*. They reported not only a statistically significantly higher prevalence of strabismus and nystagmus in these children but also that these abnormalities tend to persist at 5 years of age and are associated with long-term visual morbidity, such as lack of binocularity and poor visual acuity. The results of a study [16] on VEPs in maternal drug-exposed infants indicate that exposure *in utero* to prescribed substitute methadone is independently associated with altered Flash VEPs at least in the neonatal period. Howewer, major interest has been focused on nystagmus and the underlying mechanisms that are still uncertain. Reported studies have some limitations: i) they are based on a retrospective design [12-14], therefore including only subjects who have developed visual disorders and ii) there are difficulties in enrolling subjects whose mothers used only methadone and no other substances during pregnancy. Recently, it has been reported that, among a cohort of methadone-exposed children referred to a paediatric visual electrophysiology service because of known visual problems, 92% of infants who received pharmacological treatment for NAS developed nystagmus, while it was observable only in 38% of the infants who did not show a NAS severe enough [12].

Two possible mechanisms could account for the role of methadone in eliciting nystagmus. The most probable is the role of methadone (up-regulation) on μ- (opioid)-receptors that are present at the cerebellum level and in high concentrations in the thalamus, prefrontal and cingulated cortex, basal ganglia and midbrain structures, all involved in ocular movement control. An alternative explanation is linked to abnormal μ-opioid receptor binding in the developing brain-stem. Horizontal gaze steadiness is governed by a neural integrator that depends heavily on the nucleus prepositus hypoglossi and medial vestibular nucleus [17] where μ-opioid receptors are located [18] and activated by opiates and methadone. Intracellular recordings from medial vestibular nucleus cells in rats have shown that an activation of these receptors induced an inhibition of the tonic discharge of these cells [19] and such inhibition may cause a vertical tonus imbalance with a vertical upward drift typical of downbeat nystagmus.

To summarize the percentage of nystagmus is very high among infants with NAS who were treated, but rather uncommon although possible in untreated NAS infants. In the two cases described in this report (one with NAS and one without NAS) nystagmus gradually improved, and so did visual acuity.

## Cases presentation

Here we report two Italian cases of nystagmus in infants born from mothers exposed to methadone during pregnancy observed in the Vision Laboratory of IRCCS Fondazione Stella Maris in Pisa.

### Case 1

A second pregnancy female infant born at 33 weeks gestational age (she has a 5 year-old sister in good health). The mother was administered methadone (dosage 30 mg/d) during the entire pregnancy and denied use of other types of drugs except for diazepam. She used opiates, cannabinoids and cocaine till 7 years before. During all the pregnancy maternal urine samples were collected once a week and analyzed for methadone, opiate, benzodiazepines, amphetamines, cannabinoids and cocaine metabolites and were always in the normal range. The mother reported in the history that she did not use alcohol nor smoked. At birth NAS was not present. At 2 months the mother observed strange horizontal ocular movements and thought her daughter was blind. An ophthalmologic evaluation was performed and the fundus resulted normal. Cranial ultrasound revealed no abnormalities.

### 3 months

The infant showed significant pendular horizontal nystagmus with no null position. She was able to follow black/white target even if during the following there was an increase in pendular movements (nystagmus). Her visual acuity was low for age (Teller: 1.3 cy/cm i.e. 1.3 logMAR). She had no motor problems even if her movements were rather abrupt.

### 6 months

At 6 months the infant still showed a significant horizontal nystagmus, but she was able to follow a colour target over a large arc and she also began to perform vertical movements. She still had no null position for nystagmus and even if her visual acuity (measured by means of Teller acuity cards technique) improved it was still low for age (3.2 cy/cm i.e. 0.87 logMAR). Brain MRI was completely normal except for a cyst in a left temporal position.

### 12 months

The last examination revealed that the horizontal nystagmus was still present but she began to find null positions by deviating her eyes rightwards or downwards. Her visual acuity improved further and was only slightly lower than controls (9.8 cy/cm i.e. 0.47 logMAR). Her distance attention progressed to a range of one metre. She was able to sit unassisted, crawl and, with help, stand. She pronounced her first words like “mum” and “dad” and had good cognitive development.

### Case 2

A second pregnancy female infant born at 40 weeks gestational age (the first was a miscarriage). The mother was administered methadone during the entire pregnancy at a dosage of 35 mg/die whereas she denied use of other types of drugs. She used opiates and cocaine till 2 years before. The mother also referred that urine samples were collected once a week and analyzed for methadone, opiate, benzodiazepines, amphetamines, cannabinoids and cocaine metabolites and were always in the normal range. The use of alcohol, tobacco or other types of substances during pregnancy was also denied.

Other information about the mother were not available for her lack of cooperation. At birth NAS was present and treated with phenobarbital for 15 days. Starting from the first weeks a difficulty in the visual following was noticed, especially in the right hemifield.

### 5 months

Parents noticed abnormal ocular movements and nystagmus often associated with frequent abnormal movements involving all four limbs (similar to startle). The child was then hospitalized and examinations were performed. EEG, cranial ultrasound and ophthalmological evaluation were all within normal range. Flash VEPs were normal except for increase latency and low amplitude in right eye of non-specific significance. Brain MRI showed a venous angioma near the lateral portion of the left ventricle not correlated with the ocular disorder. The neurological development was normal for age.

The visual function examination revealed a pendular horizontal nystagmus with fixation that worsened when the child moved her eyes towards the right hemifield. Visual following was difficult with repeated loss of target. Horizontal saccades were present while vertical saccades were difficult because when the child tried to follow a vertically moving target nystamus increased and a rotative component was also noted. The visual acuity was low for her age (3,2 cy/cm i.e. 0.87 logMAR). No reductions in the visual field were found.

### 8 months

An improvement of visual function was observed. The infant still showed horizontal pendular nystagmus but less invalidating. She was able to follow a target over a large arc also vertically. Visual acuity was still low for her age (9,8 cy/cm i.e. 0.47 logMAR) but improved. Neurological development was normal.

## Conclusion

There are only a few studies in literature that question the existence of a relationship between congenital nystagmus and methadone during pregnancy and, to our knowledge, this is the first description for the Italian population. In the first case the infant did not show NAS but an additional risk factor was the use of diazepam during pregnancy that, as in other benzodiazepines, can increase the risk of nystagmus [10,20]. In the second case, instead, the infant showed NAS that is often associated with nystagmus [12]. During the first examination the infants showed different characteristics but, in both cases, there was a progressive improvement of the ocular disorder, a persistent pendular horizontal nystagmus and almost normal range visual acuity.

These two cases and other studies recently published on this topic suggest paying careful attention to prenatal history of children born with congenital nystagmus in absence of other etiopathogenesis factors. Particular regard must be focused on substances and drugs that mothers could have taken during pregnancy, the dosages and frequencies. The observed differences among subjects might be due to differences in dosage or to different combinations of substances and/or drugs. When confronted with infants with congenital nystagmus, a medical history regarding pregnancy, use of substances or drugs, dosages and the start, duration and stop of drug use should be carefully investigated.

## Consent

“Written informed consent was obtained from the patient for publication of this Case report and any accompanying images. A copy of the written consent is available for review by the Editor of this journal”.

## Abbreviations

NAS: Neonatal abstinence syndrome; EEG: Electroencephalogram; VEP: Visual evoked potential; MRI: Magnetic resonance imaging.

## Competing interests

The authors declare that they have no competing interests.

## Authors’ contributions

FT and AG conceived the study and drafted the manuscript. FT evaluated all the two children. RB contributed to data acquisition and drafting of the article. GC contributed to data interpretation, to critical revision of the article and to final approval of the version to be published. All authors read and approved the final manuscript.
